# Human spatial memory implicitly prioritizes high-calorie foods

**DOI:** 10.1038/s41598-020-72570-x

**Published:** 2020-10-08

**Authors:** Rachelle de Vries, Paulina Morquecho-Campos, Emely de Vet, Marielle de Rijk, Elbrich Postma, Kees de Graaf, Bas Engel, Sanne Boesveldt

**Affiliations:** 1 Division of Human Nutrition and Health, Wageningen University and Research, P.O. Box 17, 6700 AA Wageningen, The Netherlands; 2 Consumption and Healthy Lifestyles, Wageningen University and Research, Wageningen, The Netherlands; 3 Mathematical and Statistical Methods (Biometris), Wageningen University and Research, Wageningen, The Netherlands

**Keywords:** Evolution, Psychology

## Abstract

All species face the important adaptive problem of efficiently locating high-quality nutritional resources. We explored whether human spatial cognition is enhanced for high-calorie foods, in a large multisensory experiment that covertly tested the location memory of people who navigated a maze-like food setting. We found that individuals incidentally learned and more accurately recalled locations of high-calorie foods – regardless of explicit hedonic valuations or personal familiarity with foods. In addition, the high-calorie bias in human spatial memory already became evident within a limited sensory environment, where solely odor information was available. These results suggest that human minds continue to house a cognitive system optimized for energy-efficient foraging within erratic food habitats of the past, and highlight the often underestimated capabilities of the human olfactory sense.

## Introduction

A recurring fitness-relevant task faced by all species is the efficient pursuit of nutritional resources^1^. A central theorem of optimal foraging theory is that an individual’s fitness is a direct function of the efficiency with which one acquires energy, and natural selection pressures favour foraging traits that maximize the net rate of energy gain^1,2^. Although this theory has been extensively referenced in relation to the foraging strategies of other animals^2^, the question of whether humans also inherently carry adaptations geared toward energy-efficient foraging has not been thoroughly assessed to date.

For about 99 percent of human evolution, our ancestors were hunter-gatherers inhabiting a highly complex and variable physical food environment, where food sources varied on both spatial and temporal availabilities^3,4^. A cognitive adaptation that could have evolved to optimize foraging efforts within such erratic food habitats of the past is a high-calorie bias in spatial memory^4,5^. Such an inbuilt spatial bias entails the automatic registration and prioritization in memory of high-calorie food locations. This would have enabled foragers to efficiently navigate toward valuable calorie-dense resources – without competing for limited attentional capacities required in other important activities such as avoiding predation^4,6^. Indeed, a similar mechanism has been observed in other animal species^7–9^. Using an innovative and ecologically valid experimental set-up that covertly tested the food location memory of more than 500 individuals, we provide first-hand evidence that human spatial processing is implicitly biased toward high-calorie foods.

**Figure 1:**
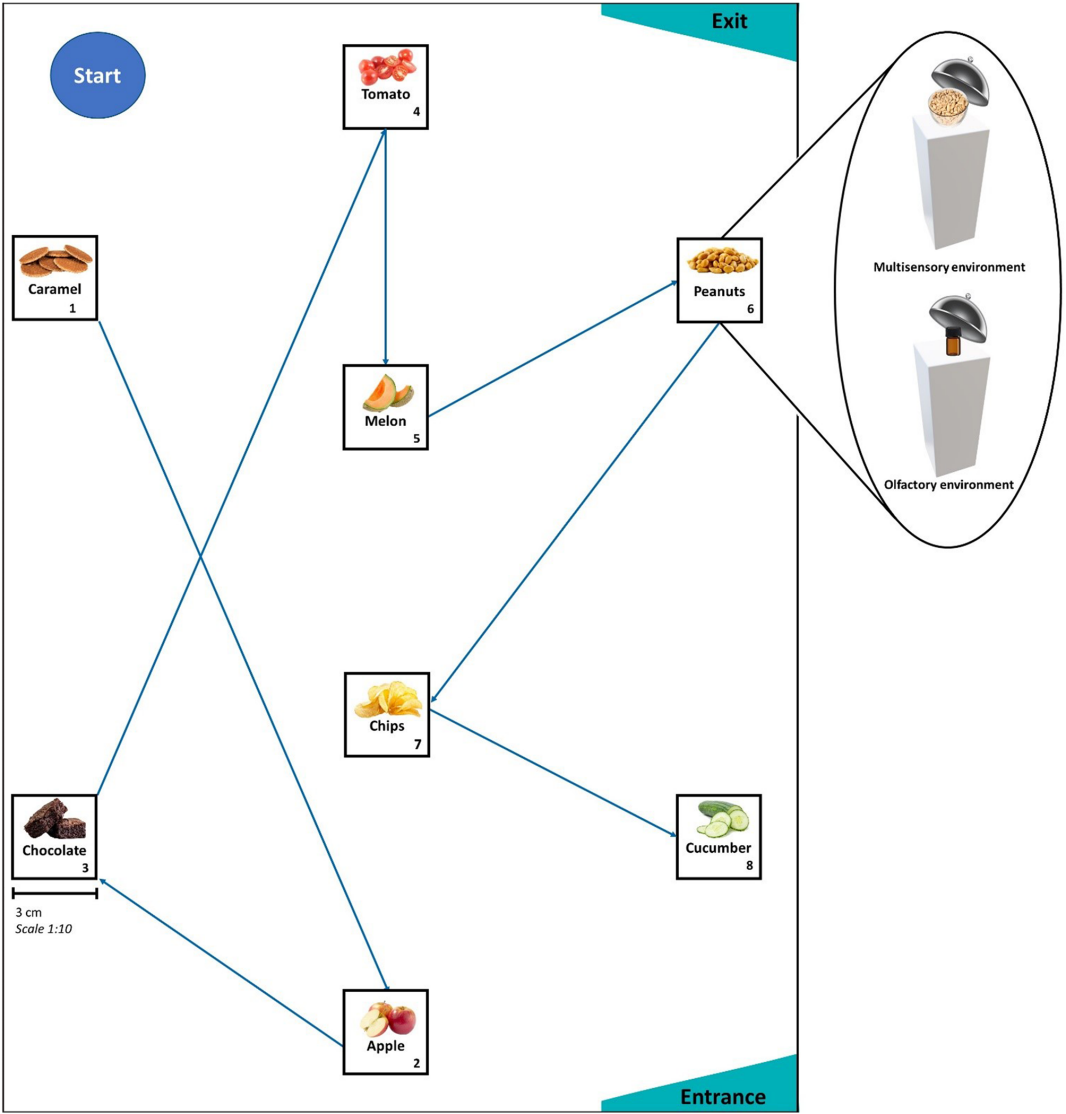
Heterogeneous food environment. Example of the spatial distribution of food stimuli and navigation route within the maze-like experimental setting.

To mirror real-world navigation within a heterogeneous food environment as closely as possible, we created a maze-like setting where participants followed a specific route within a room to sample an assortment of (sweet and savory) high- and low-calorie food stimuli at dispersed pillar locations (Fig. 1). We emulated two sensory environments in separate rooms, each of which engaged sensory modalities fundamental to the processes of spatial navigation and eating behavior^10–12^: In the *multisensory environment* (i.e. vision + taste + olfaction), stimuli consisted of actual food products that individuals had to eat, whereas individuals were instructed to only smell food odors in the *olfactory environment*. Importantly, participants were not informed that their (spatial) memory would be tested afterwards, to ensure that the encoding of food locations would be purely incidental. We then compared performance, expressed as the proportion of correct food-to-pillar relocations in a surprise spatial memory task, for high-calorie versus low-calorie food stimuli in both sensory environments.

## Results

### Human spatial memory automatically prioritizes high-calorie food

**Figure 2:**
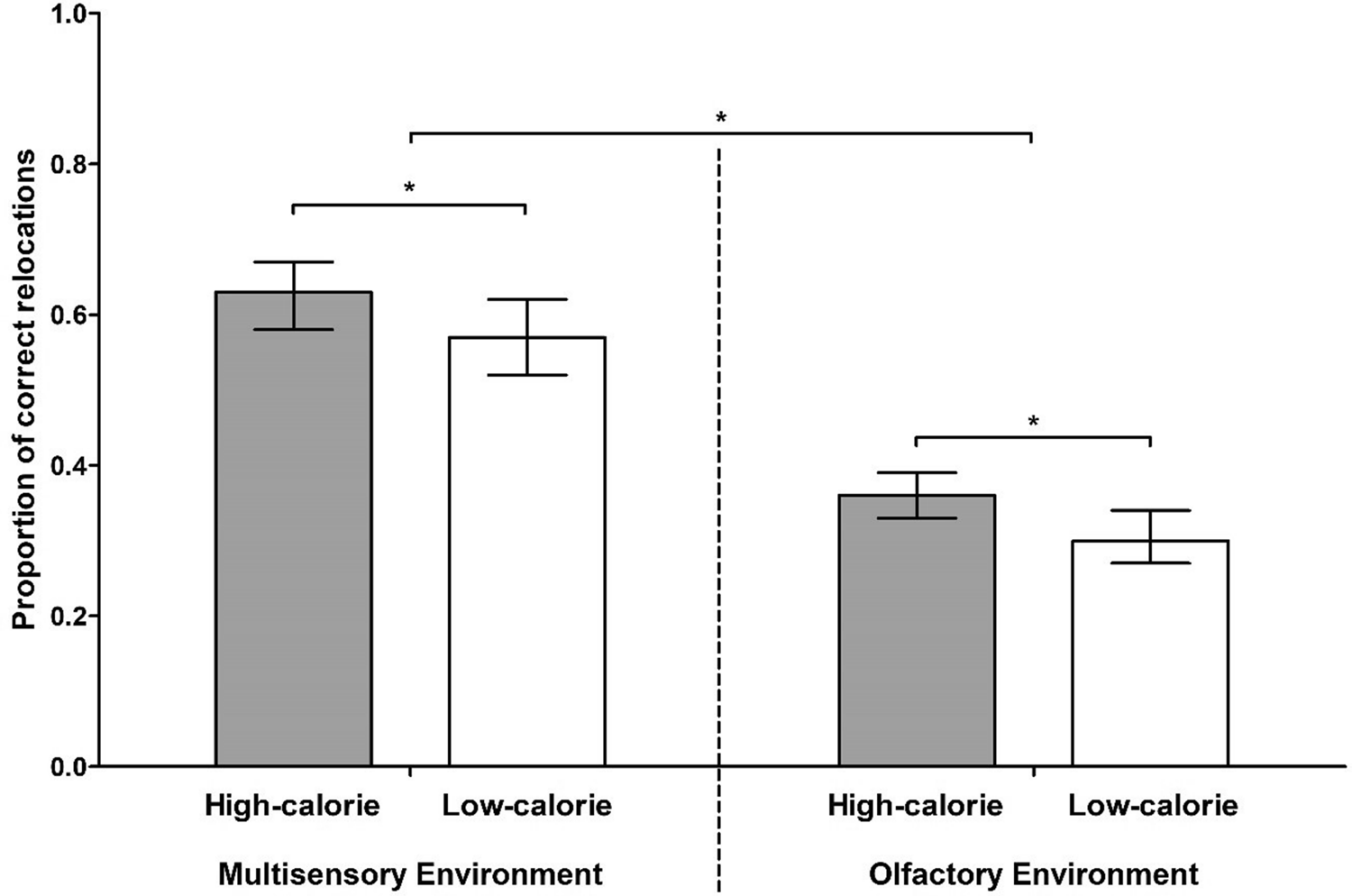
Food spatial memory accuracy. Human spatial memory for high-calorie and low-calorie food stimuli in two sensory environments, expressed as the proportion of correct food-to-pillar relocations. Error bars represent 95% confidence intervals.

**Figure 3:**
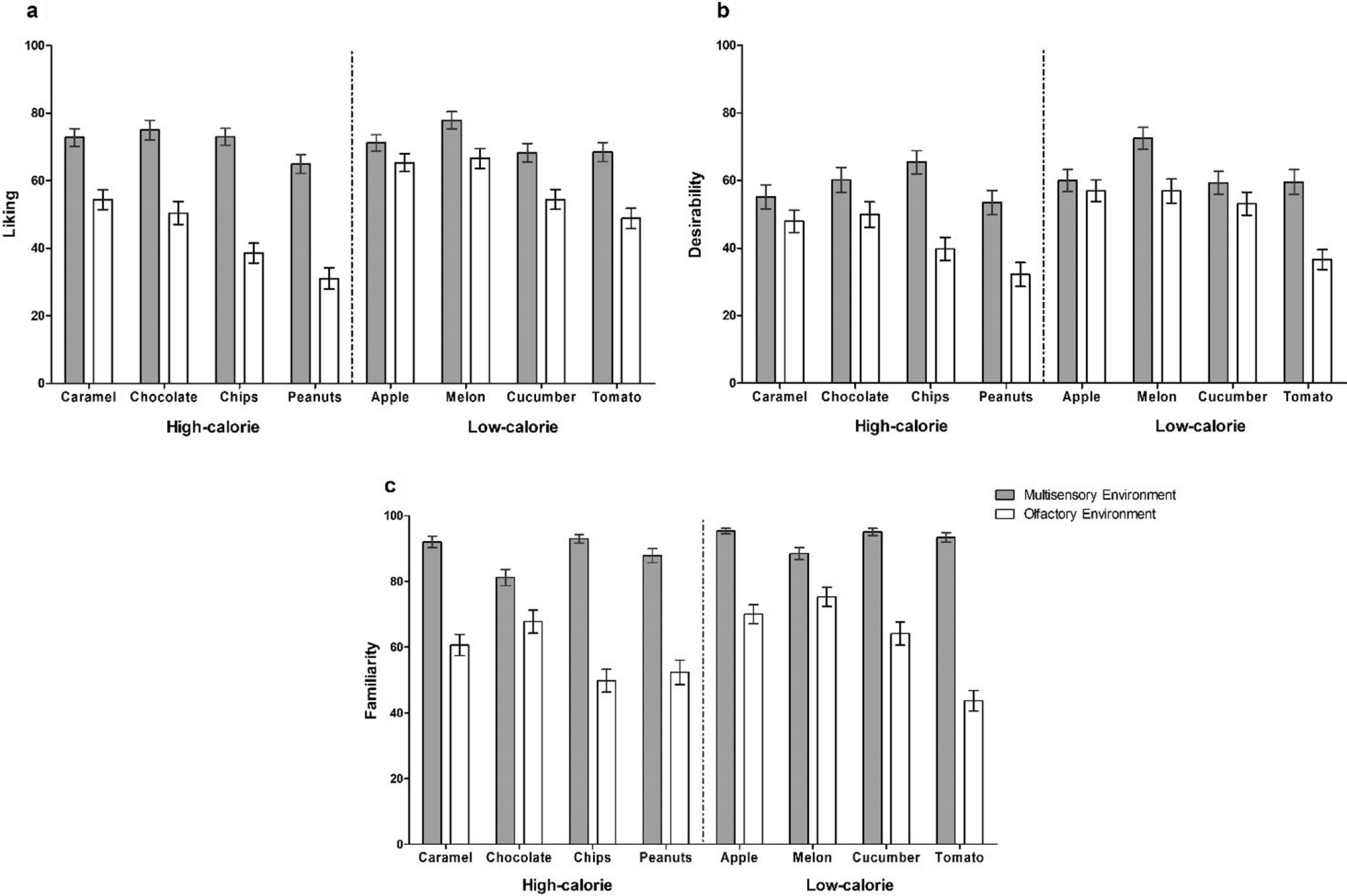
Food ratings across sensory environments. Liking (**a**), Desirability (**b**), and Familiarity (**c**) ratings (on a 100 mm Visual Analogue Scale) for all food stimuli in the multisensory and olfactory environment. Error bars represent 95% confidence intervals.

In the multisensory environment, individuals relocated high-calorie foods to correct pillar locations significantly more frequently than low-calorie alternatives (High-calorie: *M* = 0.63, 95% CI = [0.58,0.67]; Low-calorie: *M* = 0.57, 95% CI = [0.52,0.62]), χ^2^ (1) = 9.35, *p* = 0.002, OR = 1.27, 95% CI = [1.09, 1.48] (Fig. 2). This effect occurred regardless of demographics, relevant state characteristics (e.g. hunger and alertness), hedonic evaluations of foods (i.e. liking and desirability ratings; Fig. 3), and familiarity with foods. Similarly, individuals in the olfactory environment more frequently relocated odors signaling high-calorie foods to correct pillar locations relative to low-calorie odor counterparts (High-calorie: *M* = 0.36, 95% CI = [0.33,0.39]; Low-calorie: *M* = 0.30, 95% CI = [0.27,0.34]), χ^2^ (1) = 6.88, *p* = 0.009, OR = 1.28, 95% CI = [1.06, 1.54] (Fig. 2), while controlling for the same set of potential confounders – although the likelihood of a correct relocation increased with a greater familiarity with an odor stimulus, χ^2^ (1) = 47.31, *p* < 0.001, OR = 3.55, 95% CI = [2.47,5.09]. Conversely, spatial memory accuracy did not vary according to the taste of a food (i.e. sweet or savory) in either sensory condition.

### The high-calorie bias in human spatial memory manifests with limited sensory information

In a combined analysis of both sensory conditions, a better *overall* food relocation performance was observed in the multisensory compared to the olfactory environment (Multisensory: *M* = 0.58, 95% CI = [0.54,0.61]; Olfactory: *M* = 0.36, 95% CI = [0.33,0.39]), χ^2^ (1) = 62.95, *p* < 0.001, OR = 2.43, 95% CI = [1.95,3.03], after adjusting for differences between participant samples (Fig. 2). However, the sensory nature of food stimuli did not moderate the effect of caloric density on spatial memory accuracy,χ^2^ (1) = 0.49, *p* = 0.486, indicating that the high-calorie spatial memory bias was equally expressed in both sensory environments – even where solely odor information was available.

## Discussion

In a naturalistic multisensory experiment, individuals incidentally learned and more accurately recalled locations of high-calorie food stimuli. These results are compatible with the notion of “adaptive memory”, which contends that memory systems – much like other biological systems – were shaped by the forces of natural selection and should therefore show sensitivity to fitness-relevant content^13,14^. Indeed, alternative interpretations of our findings that are grounded in more traditional memory frameworks, which champion the primacy of content-insensitive general learning mechanisms, can be ruled out by our data^13^. The possibility that the high-calorie spatial memory bias resulted from a greater “depth” of processing or motivational salience of high-calorie stimuli is minimal, given that we controlled for an individual’s personal familiarity with a food, as well as their explicit liking and desire to consume an item^15^. In addition, high- and low-calorie food products were equivalent in their composition of important macronutrients (i.e. protein to carbohydrate and fat ratios), rendering it unlikely that differences in nutritional balance – rather than caloric content – is what drove the mnemonic advantage in the high-calorie condition^16^. However, the observation that (odor) familiarity predicted a higher frequency of overall correct relocations illustrates the importance of considering *both* content-sensitive and content-insensitive learning processes for human spatial cognition^5^.

Remarkably, the expression of the high-calorie bias in human spatial memory required only a limited presence of sensory information – granted that available sensory cues (such as odors) can communicate the relative value (e.g. caloric content) of potential foods – which further speaks to the processing efficiency of the mechanism^1,17^. We speculate that this could be due to an overlap in underlying (hippocampal) neural coding processes, despite variations in the (dominant) sensory modality used to explore the external world and significant objects contained within them^18^. For instance, it is feasible that hippocampal place cells show enhanced activity during recognition of objects (or cues) that flag a high-priority resource, independently of the type of sensory input received^18^. However, a sizeable difference in *overall* spatial memory performance was evident between sensory conditions, which may have resulted from a greater variety of sensory information present in the multisensory environment. Individuals in the multisensory environment had a wider availability of sensory modalities (e.g. visual information) to utilize as spatial cues during encoding, which could have yielded a richer construction of mental spatial representations^19,20^. Going forward, research efforts would benefit from additionally documenting or matching participant samples on individual abilities to mentally represent and flexibly manipulate spatial information (i.e. between the viewer-centered perspective during navigation and the aerial map perspective during spatial recall)^21^, for a more refined comparison of (food) location memory between sensory conditions*.*

In turn, differences in the expression of the high-calorie spatial memory bias may offer a novel explanation for why some individuals are less successful in maintaining a healthy energy balance within the modern food landscape^22^. An enhanced memory for high-calorie food locations could make high-calorie options relatively easier to obtain within a diverse food environment, especially for those with a greater expression of the bias^22^. In this manner, the cognitive bias may facilitate high-calorie food choice, by capitalizing on the tendency of individuals to prefer convenient easily-accessible items when making food decisions^23^. Similarly, it could stimulate individuals to visit calorie-laden food locations (e.g. fast food outlets) on a wider scale of space. Given the paucity of literature on the high-calorie spatial memory bias and its potential behavioral effects, further investigation is merited on what other cognitive processes are associated with the bias, and how it may influence the manner in which people navigate contemporary food replete settings.

Finally, our findings add to a growing literature that highlight the relevance of olfaction for eating behavior in humans, which is known to be the case across other species^11,12^. The human sense of smell is often depicted to be inferior to those of other mammals, such as dogs or rodents^24^. However, our observations showcase the intact ability of individuals to distinguish different odor types, deduce caloric properties of signaled foods from odor cues, and localize odor objects in space^11,17,25^. Indeed, a well-developed olfactory sense is thought to have conferred a survival advantage to (ancestral) hunter-gatherers^26,27^.

Taken together, we find that human minds may continue to house an implicit cognitive system optimized for energy-efficient foraging within the fluctuating ancestral food environments in which memory evolved.

## Materials and methods

### Participants

This experiment was part of the three-day Lowlands Science 2018 festival program (the Netherlands). A total of 512 attendees were analyzed: 258 participants (47% female; *M*_Age_ = 28.2 years, *SD* = 9.1; *M*_BMI_ = 24.0 kg/m^2^, *SD* = 3.6) in the multisensory environment and 254 participants (50% female; *M*_Age_ = 28.5 years, *SD* = 9.0, *M*_BMI_ = 23.8 kg/m^2^, *SD* = 3.4) in the olfactory environment. Data from 539 individuals were initially collected, but 21 files contained missing values and 6 files originated from individuals who participated in both sensory conditions which was an exclusion criterion. All participants (and/or their legal guardians) provided written informed consent prior to testing. This study was approved by the Social Sciences Ethics Committee of Wageningen University and was performed in accordance with relevant ethical guidelines and regulations. The hypothesis, full research protocol and analysis plan were preregistered, and can be accessed alongside reported data at https://osf.io/2rwmt/.

### Spatial memory task

Participants were brought to a starting point within a room (area of 12 m^2^). They navigated between eight pillars at a fixed pre-determined order that was indicated by arrow signs on the floor. Although navigation schemes remained constant, the assignment of food stimuli to pillar locations (i.e. encoding order of caloric density—taste conditions) was randomized every hour and pillar frequencies did not differ between conditions. Participants tasted (or smelled) and provided ratings (i.e. liking, desire to eat, familiarity; Fig. 3) on a food stimulus at all pillars. Participants then completed a surprise spatial memory task in a separate area. During recall, participants were randomly presented with a sequence of previous food stimuli and had to indicate the pillar location of each item on a (two-dimensional) digital map of the relevant room. The total number of possible pillar locations (N = 8) was displayed anew each recall round, and a pillar location could be selected more than once.

### Food stimuli

Four high-calorie (*M* = 498.5 kcal/100 g, *SD* = 35.8) and low-calorie (*M* = 34.3 kcal/100 g, *SD* = 18.9) food products and odor equivalents were used, with an equal number of sweet (e.g. High-calorie: chocolate brownie; Low-calorie: apple) and savory (e.g. High-calorie: potato chip; Low-calorie: cherry tomato) options for each. Food odors were matched on perceived intensity (i.e. 55–75 mm on a 100 mm Visual Analogue Scale) between caloric density—taste conditions and validated in previous research^5^. Food products were placed in bowls and refilled at regular time intervals to maintain a consistent presentation volume. Food odors were presented in (screw-capped) brown bottles (50 ml) containing scented cotton pads, which participants had to first open in order to smell. Odor bottles were also replaced regularly to uphold the desired odor intensity. All food stimuli were placed atop pillars and covered by identical cloches that participants had to open during navigation.

### Statistical analysis

For data from each sensory environment, a generalized linear mixed model (GLMM) with a random slope was formulated. A GLMM was chosen to flexibly model for correlated errors in the (non-normal) binary outcome variable^28^, and linearity of covariates (on the logit scale) was shown to sufficiently capture their effects. The GLMM comprised fixed main and interaction effects for experimental factors *Caloric Density* and *Taste*, and random effects for the factor *Participant*. All effects were introduced on the logit scale. Additionally, in the fixed part of the model and also on the logit scale, *Gender*, *Age* (in tertiles), *Subjective SES*, *Food Allergies*, *Hunger* ratings, hours of *Sleep*, *Alertness*, *Alcohol consumption, Drug use*, *Smoking, Liking*, *Desirability*, and *Familiarity* were entered as covariates. Binary observations, conditional upon the random effects for participants, were assumed to follow a Bernouilli distribution. To test whether the type of sensory environment (i.e. multisensory versus olfactory) moderates food spatial memory accuracy and expression of the high-calorie bias, observations from both sensory rooms were combined into a single analysis, adding fixed main and interaction effects (e.g. with *Caloric Density*) of *Sensory Environment* to the GLMM. Ordinary likelihood ratio tests (using the -2LL test statistic) were used for testing, with *p* values derived from an approximation with the chi-square distribution. Inference was based on Laplacian integration employing the *lme4* package from R^29^. Detailed information on the measurement of covariates and the model selection process can be found at https://osf.io/2rwmt/.

## Data Availability

The data that support the findings of this study are available on the Open Science Framework repository with the identifier 10.17605/OSF.IO/2RWMT^30^.
